# *Mycobacterium tuberculosis* infections in cynomolgus monkey transplant recipients and institution of a screening program for the prevention and control of tuberculosis

**DOI:** 10.1186/s12917-016-0898-y

**Published:** 2016-12-20

**Authors:** Eun Wha Choi, Kyo Won Lee, Tae Min Kim, Hyojun Park, Mi Ri Jeon, Chan Woo Cho, Jae Berm Park, Sungjoo Kim

**Affiliations:** 1 Laboratory Animal Research Center, Samsung Biomedical Research Institute, 81 Irwon-ro, Gangnam-gu, Seoul 135-710 Republic of Korea; 2 Department of Surgery, Division of Transplantation, Samsung Medical Center, Sungkyunkwan University School of Medicine, 81 Irwon-ro, Gangnam-gu, Seoul 135-710 Republic of Korea; 3 Transplantation Research Center, Samsung Biomedical Research Institute, Samsung Medical Center, Seoul, Republic of Korea

**Keywords:** Mycobacterium tuberculosis, Cynomolgus monkey, Nonhuman primate, Acid-fast bacilli, Transplantation, Tuberculin skin testing

## Abstract

**Background:**

Tuberculosis is a major health concern in not only humans, but also in non-human primates. In this paper, we report recent cases of *Mycobacterium tuberculosis* in cynomolgus monkeys from Cambodia used in transplantation research in a Korean facility and describe a program instituted to prevent and control subsequent infections.

**Case presentation:**

All monkeys were antibody negative for tuberculosis during quarantine; however, suspected tuberculosis gross lesions were observed in two cynomolgus monkeys who underwent allograft kidney transplantation. Lung tissue from one monkey was found to be weakly positive by PCR for detection of *M. tuberculosis*. After PCR confirmation of tuberculosis, we decided to sacrifice the remaining animals and instituted a program for preventing subsequent infections. During necropsy of the remaining monkeys, two additional suspected tuberculosis cases were observed. A total of four monkeys with nodular lesions in the respiratory tract, suspected to be tuberculosis, demonstrated no clinical signs. Acid-fast bacilli were identified on slides from the lung or liver in all four monkeys. Two of four monkeys tested PCR positive. We decided that new monkeys entering from Cambodia should undergo a single gastric aspiration PCR and tuberculin skin testing (TST) every 2 weeks until four consecutive negatives to detect latent tuberculosis are obtained before starting experiments. Monkeys should then undergo a chest X-ray monthly and TST every 6 months.

**Conclusions:**

Detection of latent tuberculosis by an effective preventive screening program before starting experiments is an essential process to reduce the risk of reactivation of tuberculosis, especially in studies using immunosuppressive drugs. It also serves to protect the health of captive non-human primates, their caretakers and researchers.

## Background

Tuberculosis is responsible for more than 2 million deaths worldwide each year [1] and is the second most common cause of infectious disease-related death. Roughly one-third of the world’s population has been infected with *Mycobacterium tuberculosis*, but the majority of infections are latent and only ~10% of those infected progress to active tuberculosis [2, 3]. Tuberculosis is a common bacterial infection with a high incidence in developing countries. The distribution of tuberculosis is not uniform across the world; of the 9.6 million new tuberculosis cases in 2014, 58% were in Southeast Asia and the Western Pacific regions, according to a WHO report [4]. Old World monkeys can maintain latent tuberculosis infections [3, 5], but there are very few published studies on the prevalence of mycobacterial infections in non-human primates. According to one study, the overall prevalence of M. tuberculosis complex as determined by polymerase chain reaction (PCR) from buccal samples was 31.9% of the macaques in four Asian countries and Gibraltar [6].

Non-human primates are needed for i) safety testing of pharmaceuticals, ii) research on infectious diseases such as HIV, hepatitis C, malaria and tuberculosis [7–9] iii) research on the human brain [10], and iv) research on organ transplantation. With regard to organ transplantation, the shortage of donors for organ transplantation and the limited organ supply are major problems [11]. The pig represents the most likely source animal candidate; however there are serious immunological incompatibilities between pigs and primates based on a specific immune response due to anti-α-gal. Studies on primates are also needed to determine therapeutic regimens and to evaluate new immunosuppressants such as monoclonal antibodies to certain proteins that may be shared by humans and monkeys.

A tuberculosis outbreak in a non-human primate facility is a serious threat to the health of non-human primates as well as their caretakers and researchers [12].

In this paper, we report recent cases of *M. tuberculosis* in cynomolgus monkeys (*Macaca fascicularis*) from Cambodia in the zone for transplantation research in a Korean facility and the program instituted to prevent and control subsequent infections.

## Case presentation

All experimental monkeys were from an animal supplier for medical research (ORIENT CAM CO., LTD., Kampong Chhnang, Cambodia). All cynomolgus monkeys underwent tuberculin skin testing (TST) semiannually in Cambodia before importation. Monkeys were imported for transplantation studies. Transplantation studies were conducted by the researchers of Samsung Medical Center at the facility located in Genia Inc. (Sungnam, Republic of Korea). Blood samples from all monkeys were PCR negative for herpes B virus, simian immunodeficiency virus, simian T lymphotropic virus, simian retrovirus type D and tuberculosis. These tests were performed by a commercially available animal diagnostic laboratory (Zoologix Inc., Chatsworth, CA, USA). During quarantine, all monkeys also received tuberculosis (TB) antibody tests (SD Rapid TB, Standard Diagnostics, INC., Yongin, Korea), and the results of these were all negative. All monkeys were singly housed in stainless steel caging in a room maintained at 23 ± 3 °C (June –August: 25 ± 4 °C) with 30–70% humidity. All studies of allo-kidney transplantation and allo-islet transplantation using cynomolgus monkeys were approved by the Institutional Animal Care and Use Committee (IACUC) of the Genia Inc. (IACUC number; ORIENT-IACUC-16018 and ORIENT-IACUC-16019, respectively). All procedures were in compliance with Animal Welfare Act Regulations and the Guide for the Care and Use of Laboratory Animals. The facility has been audited by Korean Quarantine Inspection Agency and Ministry of Food and Drug Safety.

### Case #1

**Fig. 1:**
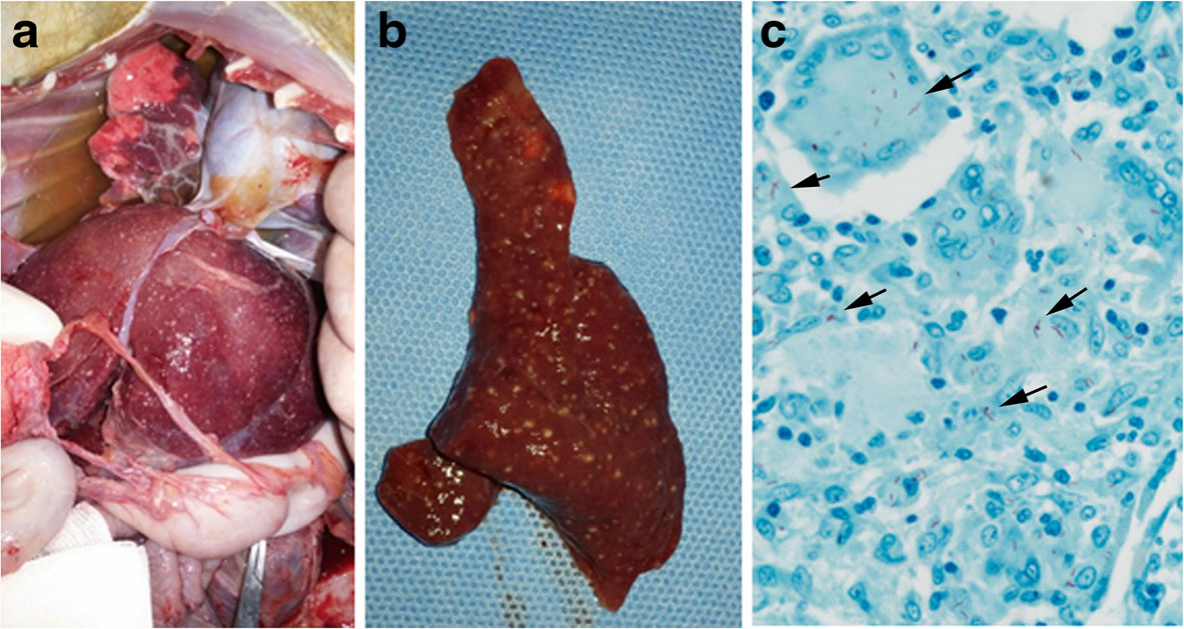
Gross and histological lesions from a cynomolgus monkey who underwent allograft kidney transplantation suspected to have tuberculosis (Case #1). **a** Miliary lesions of the liver and lung of AK-08. **b** Granulomas in the spleen of AK-08. **c** Acid-fast bacilli (*arrow*) in liver tissue (AK-08, Ziehl-Neelsen stain × 400)

A 5-year-old Cambodian male cynomolgus monkey (AK-08) was transferred to the transplantation unit following successful completion of a one-month quarantine. Allograft kidney transplantation was performed and immunosuppressive drugs (mycophenolate mofetil and cyclosporine) were administered. Native kidney nephrectomy and telemetry insertion were performed at day 13 after kidney transplantation and ureter revision was conducted due to hydronephrosis at day 28 after kidney transplantation. Kidney biopsy was done under general anesthesia at day 30 after kidney transplantation, but the monkey did not awaken from general anesthesia. He demonstrated no coughing, dyspnea, or other clinical signs localized to the respiratory tract. On necropsy, very small nodules throughout the liver and spleen and nodular lesions in the lung were observed (Fig. 1a and b). The tissues were fixed, paraffin embedded, sectioned and stained with hematoxylin and eosin (H&E) or underwent acid-fast bacilli (AFB) staining for microscopic evaluation. AFB were identified on the slide from the liver (Fig. 1c). No AFB were identified on the slides from the lung and spleen.

To further confirm the results, a genetic analysis by PCR was conducted at a diagnostic service laboratory (SCL, Yongin, Korea). Lung tissue was found to be weakly positive by PCR for detection of *M. tuberculosis* and negative by PCR for detection of non-tuberculous mycobacteria (NTM).

### Case #2

**Fig. 2:**
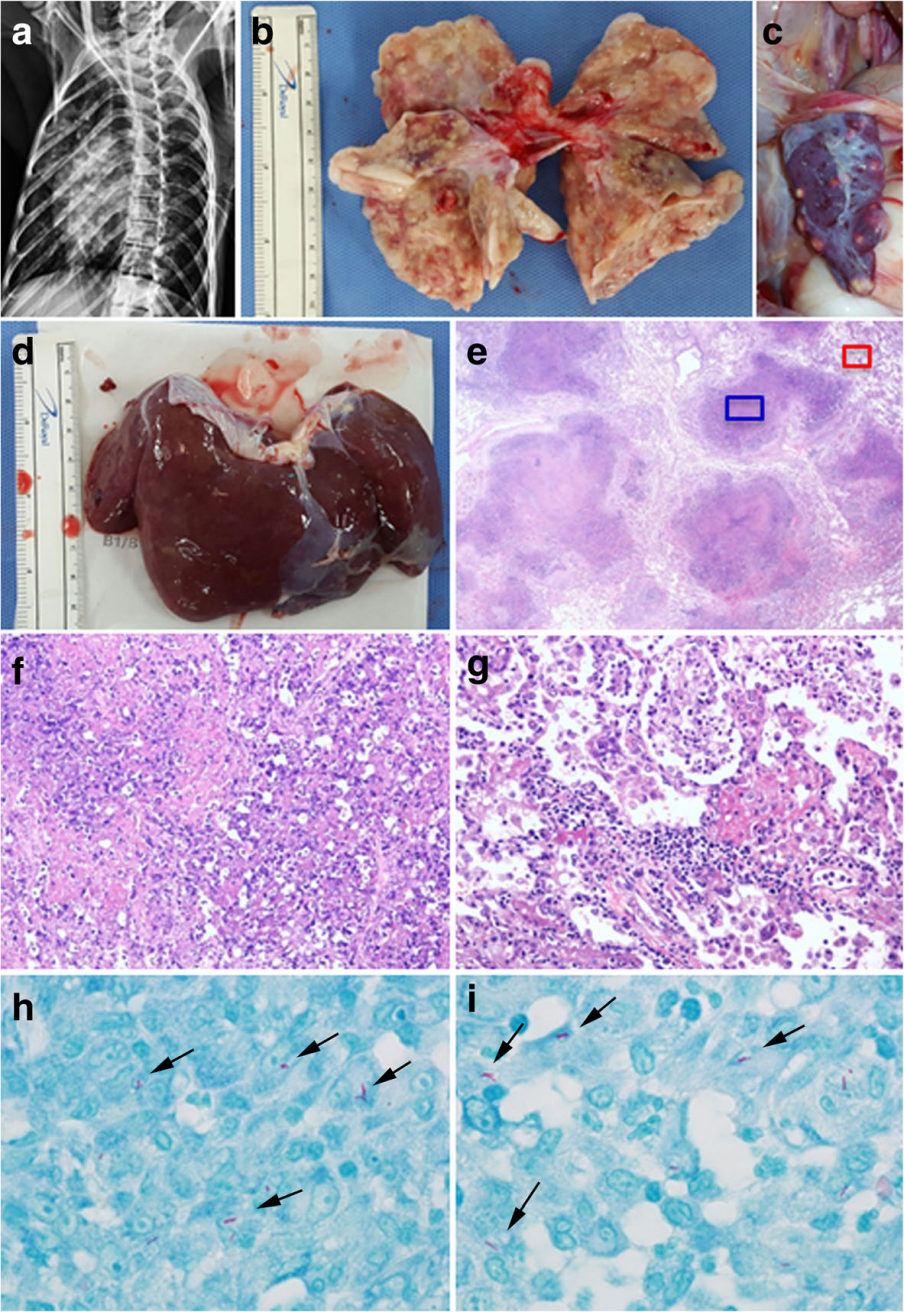
Gross and histological lesions in a cynomolgus monkey who underwent allograft kidney transplantation suspected to have tuberculosis (Case #2). **a** Chest radiograph: note the presence of infiltrate. **b** Granulomatous lung lesions from AK-09. **c** Granulomatous spleen lesions from AK-09. **d** Granulomatous liver lesions from AK-09. **e** Granuloma with central caseous necrosis in lung tissue (AK-09, H&E, ×12.5). **f** Central caseation in a granuloma, magnification of the blue area in **e** (AK-09, H&E, ×200). **g** Various inflammatory cells, magnification of the *red* area in **e** (AK-09, H&E, ×200). **h**, **i** Acid-fast bacilli (*arrow*) in lung tissue (AK-09, Ziehl-Neelsen stain × 1000)

A 5-year-old Cambodian male cynomolgus monkey (AK-09) was transferred to the transplantation unit following successful completion of a one-month quarantine. Allograft kidney transplantation was performed and immunosuppressive drugs (tacrolimus, mycophenolate mofetil and cyclosporine) were administered. Native kidney nephrectomy, graft kidney biopsy and telemetry insertion were performed at day 13 after transplantation and steroid pulse therapy was conducted due to elevated concentration of serum creatinine at day 18 after transplantation. Twenty days after transplantation, mycophenolate mofetil was stopped due to diarrhea lasting 11 days. Kidney biopsy was performed under general anesthesia at day 69 after kidney transplantation due to elevated serum creatinine (1.6 mg/dl), and rejection was confirmed on kidney biopsy. Chest radiographs were performed and pulmonary infiltrates were observed (Fig. 2a). On necropsy, nodular lesions in the lung, spleen, and liver were observed (Fig. 2b-d). Granulomatous lesions containing central caseous necrosis were observed on H&E stained lung tissue slides (Fig. 2e-g), and AFB were identified on the lung slide (Fig. 2h and i). Lung tissue samples were negative on PCR for detection of *M. tuberculosis* or NTM.

After Case#1 and Case #2 occurred, we decided to sacrifice the remaining 36 animals in the zone for transplantation research, although all had negative TB antibody test kit results (SD Rapid TB) at day 8 after PCR confirmation of tuberculosis. There are three breeding rooms in the zone for transplantation research, but all remaining animals had shared a surgery room with the affected animals. In the affected zone, entry was restricted to essential personnel only. Each person wore a uniform, an N95 mask, gloves, rubber boots, eye protection, and head covers. All animal-care staff, surgery staff and veterinary staff underwent chest X-ray and Quantiferon test (Samsung Medical Center), which demonstrated negative TB status. During necropsy of the remaining monkeys, two additional suspected tuberculosis cases were observed.

### Case #3

**Fig. 3:**
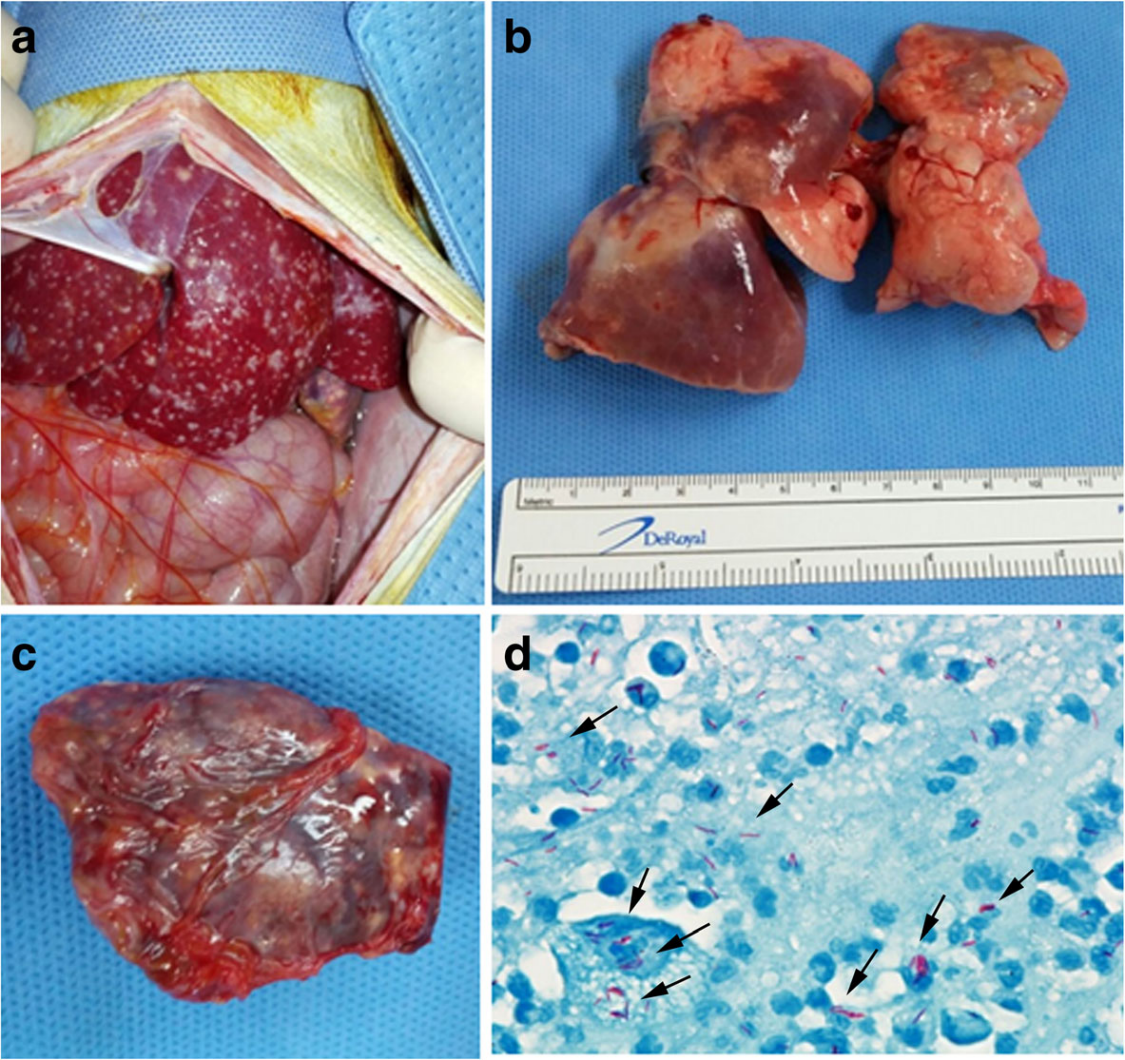
Gross and histological lesions in an additional cynomolgus monkey suspected to have tuberculosis during euthanasia (Case #3) after PCR confirmation of tuberculosis in AK-08. **a** Granulomas in the liver from 16 to 6. **b** Granulomas in the lung from 16 to 6. **c** Granulomas in the spleen from 16 to 6. **d** Acid-fast bacilli (*arrow*) in lung tissue (16–6, Ziehl-Neelsen stain × 1000)

After Case#1 and Case #2 occurred, blood samples from the 16–6 monkey (an approximately 5 year-old male) were negative for the TB antibody test and chest X-ray was performed. His general condition was not good, with reduced body weight and elevated C-reactive protein level. This monkey was sacrificed 26 days after TB antibody test. On necropsy, nodular lesions in the liver, lung and spleen were observed (Fig. 3a–c). AFB were identified on the lung slide (Fig. 3d). Sputum, lung and spleen tissue samples were PCR-positive, and the liver tissue sample was PCR-negative for detection of *M. tuberculosis*. All of these samples were PCR-negative for NTM. Mycobacteria cultures were attempted using samples from a lung abscess, lung tissue, liver tissue and blood, but negative culture results were obtained (no growth, performed at the SCL).

### Case #4

**Fig. 4:**
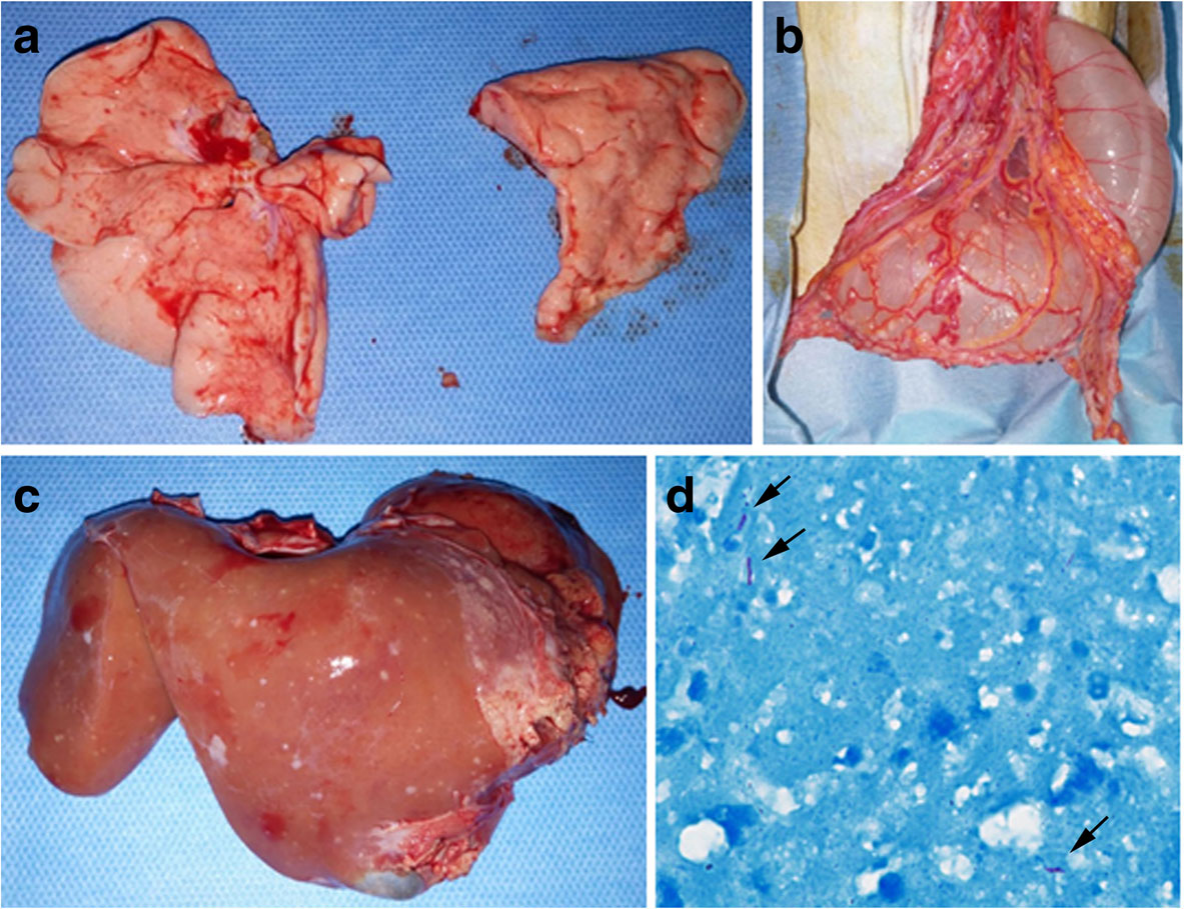
Gross and histological lesions in a cynomolgus monkey who underwent allograft islet transplantation suspected to have tuberculosis (Case #4) during euthanasia after PCR confirmation of tuberculosis in AK-08. **a** Lung from AI-42. **b** Granulomatous mesenteric lesions from AI-42. **c** Granulomatous liver lesions from AI-42. **d** Acid-fast bacilli (*arrow*) in lung tissue (AI-42, Ziehl-Neelsen stain × 1000)

Approximately 5 months before necropsy, diabetes mellitus (DM) was induced in the AI-42 monkey (an approximately 4-year-old male) by partial pancreatectomy with low-dose streptozotocin administration. Allo-islet transplantation was performed 113 days after DM modeling. Small nodular lesions were observed in the lung, liver and mesentery (Fig. 4a–c). AFB were identified on the lung slide (Fig. 4d). Lung and liver tissue samples were PCR-negative for the detection of *M. tuberculosis* or NTM.

**Fig. 5:**
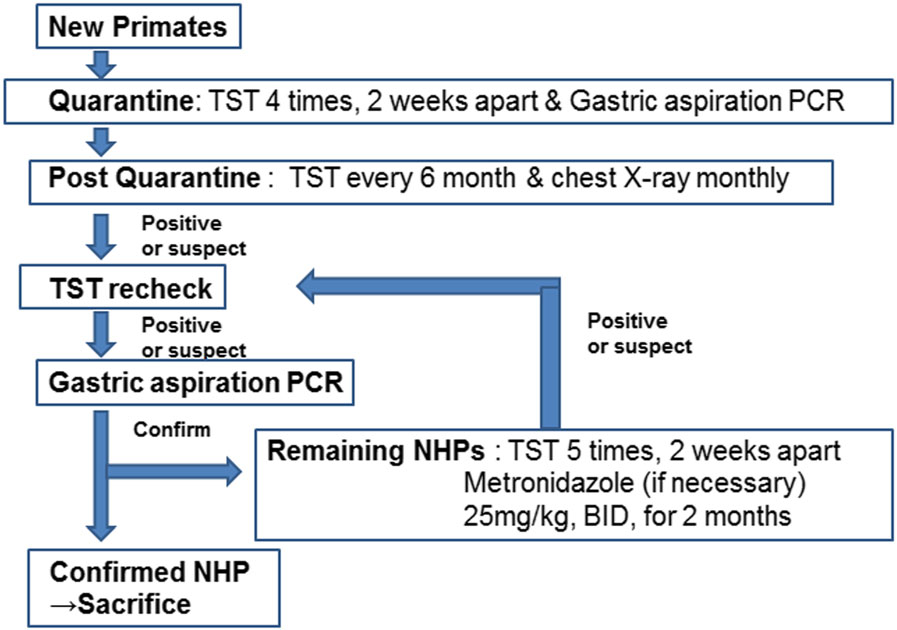
SMC screening program for the prevention and control of tuberculosis in nonhuman primates. SMC: Samsung Medical Center, TST: tuberculosis skin testing, PCR: polymerase chain reaction, NHP: nonhuman primates

At day 41 after PCR confirmation of tuberculosis (AK-08), all remaining animals were sacrificed. The zone for the transplantation study was fumigated by O2SAFE (H_2_O_2_ + C_3_H_4_O_3_) using the Minncare Dry Fog System (Mar Cor, Plymouth, MN, USA). Sterilization and cleanliness were then confirmed by a biological indicator test and microbiological evaluation. A program for preventing subsequent infections was discussed and instituted by the surgery professor staff, veterinary staff and a professor from the division of infectious diseases at Samsung Medical Center, with recommendations from the Office of Animal Care and Use (Fig. 5). We decided that new monkeys entering from Cambodia should undergo gastric aspiration PCR once and TST every 2 weeks until four consecutive negative results were obtained, in order to detect latent tuberculosis before starting experiments. Monkeys should then undergo monthly chest X-ray and TST every 6 months. Fourteen days after fumigation, new experimental monkeys were transported into the zone for the transplantation study. Nasal and gastric aspiration content samples from all new 26 monkeys were PCR-negative for detection of *M. tuberculosis* or NTM. Results from serial TSTs of the 26 new monkeys were also all negative.

## Discussion

During quarantine, all monkeys had TB antibody tests and PCR for *M. tuberculosis* with blood samples; these results were all negative. Still, suspected tuberculosis lesions were observed during the transplantation study. Four monkeys with nodular lesions localized to the respiratory tract, suspected to be tuberculosis, demonstrated no coughing, dyspnea, or other clinical signs. The severity of the respiratory signs is variable between species and strain; rhesus monkeys tend to develop overt respiratory signs more frequently than cynomolgus monkeys [12]. According to a textbook on non-human primates, monkeys often die with no relevant clinical history or clinical signs [13].

On chest radiographs from one monkey, pulmonary infiltrates were observed. According to one report, tuberculosis may present with a weak radiological contrast in non-human primates compared with other species due to the rareness of calcified tubercles [14]. A few AFB were identified on slides from the lung or liver in all four monkeys with nodular lesions. *M. tuberculosis* infection usually has very small numbers of AFB in tissue compared with *M. avium* complex infection [12]. Two of four monkeys were PCR positive and the others were PCR negative. PCR tests have a specificity close to 100%, but the sensitivity is variable [15]. Many reports revealed that transplant recipients show negative sputum smear results and negative or indeterminate TST or Quantiferon tests despite active disease and atypical clinical presentations [16]. Most monkeys in the zone for transplantation study were given immunosuppressants and underwent transplant surgery. Immunosuppression can interfere with cell-mediated immunity and may interfere with gamma interferon production (Quantiferon) and TST results. Furthermore, most of the animals shared a surgery room with infected animals. The diagnosis and selection of infected animals was very difficult in this situation. Thus we decided to sacrifice all remaining animals in the zone for the transplantation research.

Although economic growth has brought a greater national capacity for disease control, the incidence of tuberculosis in South Korea remains the highest among high-income countries [17]. All staff for the transplantation study had received the Bacillus Calmette-Guerin vaccination due to high rates of tuberculosis exposure in the general population in Korea. Because interpreting skin test results is difficult after vaccination, Quantiferon tests were used for detection of latent tuberculosis. The *M. tuberculosis* protein used in the Quantiferon test is more specific than that in the skin test because the vaccine strain does not contain these proteins [18]. No definitive source for the outbreak was identified.

TB antibody tests and PCR for *M. tuberculosis* using blood samples were not helpful in detecting latent tuberculosis in our cases. Juvenile macaques are the most susceptible to tuberculosis [14]. According to the ‘Guidelines for the Prevention and Control of Tuberculosis in Nonhuman Primates’ (oacu.od.nih.gov), TST is the primary tool used to detect tuberculosis in non-human primates and TST of Macaque species is recommended quarterly or semiannually, with a frequency of 2 weeks during quarantine and post-quarantine holding. The recommended frequency of TST in the Macaque species is quarterly or semiannually at 2-week intervals during quarantine and post-quarantine holding. The skin test can convert from a false negative to positive due to boosting in some individuals [16]. Thus serial tests at 2-week intervals can increase the detection rate of tuberculosis. After the diagnosis of tuberculosis in our study, we strengthened the screening program to prevent the occurrence of tuberculosis according to recommendations in the NIH guidelines.

Lin et al. reported that treatment with effective drugs prevents not only reactivation of latent tuberculosis infection, but also reduces bacterial load and infection spread in active tuberculosis [19]. In their study, short-term treatment with metronidazole alone (25 mg/kg bid for 2 months) reduced reactivation from latent tuberculosis infection in cynomolgus monkeys as evaluated by gross pathology, bacterial load (colony-forming unit score) and the extent of involvement and dissemination (% positive samples) [19].

Given the threat of zoonotic transmission and also high risk of spread among caregivers, a special effort should be made to implement screening procedures for humans. It is recommended that non-human primate facilities follow standard procedures and appropriate regulations (i.e., national or federal guidelines) to minimize the possibility of spread among humans or animals. For example, centers for disease control and prevention (CDC) suggests two types of testing method for health care workers; one initial test upon working in a new place, and the other on an annual basis or serial manner as determined by state regulation or risk assessment outcome. Currently we are undertaking TST or/and Quantiferon as well as chest X-ray tests on new staffs/caregivers, and all personnel receive chest X-ray test every 6 months. When TB outbreaks are suspected, all personnel are immediately screened with those two methods.

Regarding human medicine, all transplant candidates and patients who are prescribed immunomodulatory drugs such as TNF-α blockers should undergo evaluation for latent tuberculosis infection due to the high risk of occurrence (reactivation) and the difficulty in diagnosis and treatment of tuberculosis in immunosuppressive states [16].

Detection of latent TB by an effective preventive screening program before starting an experiment is essential to reduce the risk of reactivation of TB, especially in a study using immunosuppressive drugs. This also reduces the threat to the health of non-human primates, their caretakers and researchers.

It seems reasonable to expect that the new screening/management protocols described in this paper will provide a better way to identify latent carriers. However, more thorough, longitudinal follow-up studies are required to confirm the feasibility and effectiveness of these protocols under similar experimental conditions.

## Conclusions

To the best of our knowledge this is the first report of *M. tuberculosis* infection in non-human primate transplant recipients. Detection of latent TB using an effective screening program before starting an experiment is an essential component of reducing the risk of reactivation, especially in studies using immunosuppressive drugs.

## Abbreviations

AFB: Acid-fast bacilli
DM: Diabetes mellitus
H&E: Hematoxylin and eosin
NTM: Nontuberculous Mycobacteria
PCR: Polymerase chain reaction
TB: Tuberculosis
TST: Tuberculin skin testing
